# Mechanisms of Neurotoxic Symptoms as a Result of Breast Cancer and Its Treatment: Considerations on the Contribution of Stress, Inflammation, and Cellular Bioenergetics

**DOI:** 10.1007/s12609-017-0245-8

**Published:** 2017-04-22

**Authors:** Tamara E. Lacourt, Cobi J. Heijnen

**Affiliations:** Department of Symptom Research, Neuroimmunology Laboratory, The University of Texas MD Anderson Cancer Center, 1515 Holcombe Blvd, Unit 384, Houston, TX 77030 USA

**Keywords:** Cancer-related fatigue, Chemobrain, Neuropathy, Distress, Mitochondria

## Abstract

**Purpose of Review:**

Breast cancer and its treatment are associated with a range of neurotoxic symptoms, such as fatigue, cognitive impairment, and pain. Although these symptoms generally subside after treatment completion, they become chronic in a significant subset of patients. We here summarize recent findings on neuroinflammation, stress, and mitochondrial dysfunction as mechanistic pathways leading to neurotoxic symptom experience in breast cancer patients and survivors.

**Recent Findings:**

Neuroinflammation related to stress or cancer treatment and stress resulting from diagnosis, treatment, or (cancer-related) worrying are important predictors of a neurotoxic symptom experience, both during and after treatment for breast cancer. Both inflammation and stress hormones, as well as cancer treatment, can induce mitochondrial dysfunction resulting in reduced cellular energy.

**Summary:**

We propose reduced cellular energy (mitochondrial dysfunction) induced by inflammation, oxygen radical production, and stress as a result of cancer and/or cancer treatment as a final mechanism underlying neurotoxic symptoms.

## Introduction

**Fig. 1:**
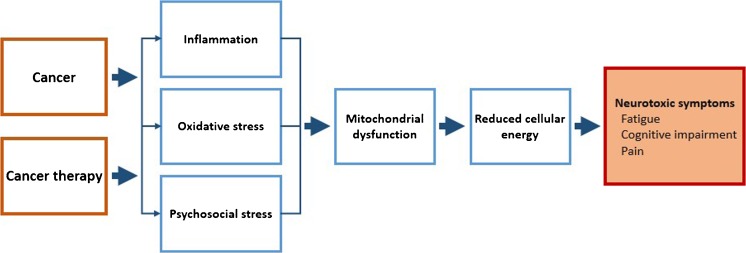
Schematic overview of proposed mechanisms underlying cancer-related and cancer therapy-related neurotoxic symptoms

Breast cancer and its treatment are associated with a range of neurotoxic symptoms, including fatigue, cognitive impairment, and pain [1–3]. The experience of neurotoxic symptoms is one of the foremost reasons for treatment reductions [4, 5], affecting treatment efficacy and, consequently, survival. In breast cancer survivors, neurotoxic symptoms negatively affect quality of life and often prevent individuals from resuming precancer activities [6]. The number of breast cancer survivors has increased exponentially over the last decades [7]. Thus, understanding the mechanisms underlying neurotoxic symptoms is fundamental for developing interventions that allow patients to tolerate necessary cancer treatments and survivors to live a life not marked by ongoing debilitating symptoms (Fig. 1).

It is now well-known that chemotherapy has severe neurotoxic side effects that can persist long after treatment completion [8–10]. Additionally, although the neurotoxic effects of radiation and surgery are less studied, the evidence for short- and long-term neurotoxic side effects from these treatment modalities is compelling [11–14]. Less well-established are the neurotoxic symptoms associated with cancer itself, present prior to commencement of any cancer treatment. Nevertheless, accumulating evidence indicates that a significant number of treatment-naïve breast cancer patients already suffer from mild cognitive deficits, fatigue, and (breast) pain [15, 16].

Neuroinflammation has been suggested as one of the main drivers of neurotoxic symptoms [17–19], although published effect sizes are moderate at most and conflicting results have been reported. Psychosocial stress is highly prevalent in breast cancer patients and can increase inflammation. Therefore, stress is considered a likely contributor to neurotoxic symptoms [19, 20]. Perceived stress typically activates the neuroendocrine system, resulting in secretion of stress hormones like cortisol and (nor)epinephrine. Neuroendocrine-immune interaction is of utmost importance for homeostasis. The homeostatic balance can be disrupted by prolonged exposure to stress. A systemic increase in cortisol as a response to an acute stressor can suppress inflammation via binding to glucocorticoid receptors (GRs) on leukocytes or other cytokine-producing cells like endothelial cells. However, when stress becomes chronic, GRs are downregulated via an intracellular process called receptor desensitization, which ultimately leads to enhanced immune responses [21, 22]. Thus, stress can contribute to neurotoxic symptom experience via an inflammatory pathway. Stress can produce symptoms via non-inflammatory pathways as well: most cells, including neurons, have receptors for stress hormones, and evidence from animal models suggests that stress-induced receptor desensitization can affect neuronal function [23].

We here review recent literature on neuroinflammation and stress in relation to neurotoxic symptom experience in breast cancer patients and survivors. We also discuss recent findings suggesting that mitochondrial dysfunction is a final common step in the effects of cancer and cancer therapy on neurotoxic symptom development.

## Neuroinflammation and Symptom Experience

Several excellent reviews have summarized findings on the association between peripheral markers of inflammation, as a proxy for neuroinflammation, and the experience of neurotoxic symptoms during and after cancer treatment [10, 17, 24, 25]. Recent additions to these reviews are discussed below.

To the best of our knowledge, the association between inflammation and neurotoxic symptoms in *treatment-naïve* breast cancer patients has been reported in only one study. Patel et al. [26••] found that in treatment-naïve breast cancer patients, but not in controls, higher plasma levels of soluble TNF receptor 2 (sTNFR2) were associated with heightened memory impairment. However, sTNFR2 was not higher in patients relative to controls, suggesting that breast cancer patients might be more susceptible to the effects of inflammatory mediators even before treatment.

### Neuroinflammation and Symptoms During Cancer Therapy

Cancer therapy can elicit an inflammatory response through several pathways, including direct immune changes in the tumor microenvironment, tumor cell death, and damage to healthy tissue [27–32]. Associations between symptoms and inflammatory markers such as interleukin (IL)-6, tumor necrosis factor (TNF)-α, and C-reactive protein (CRP) have been observed for every treatment modality, in both cross-sectional and longitudinal designs [13, 33–38]. Smith et al. [39] observed reduced DNA methylation in blood mononuclear cells in patients who recently completed chemotherapy (compared with treatment-naïve patients) that was associated with higher plasma concentrations of sTNFR2 and IL-6. sTNFR2 in turn was associated with fatigue. Interestingly, these associations were only observed shortly after chemotherapy completion, suggesting a transient effect of chemotherapy on inflammation and subsequent fatigue.

Associations between inflammatory markers and symptom severity are not always driven by cancer treatment. For example, Reed et al. [40] observed an increase in plasma TNF-α as well as in the severity of a cluster of symptoms (including fatigue, nausea, and pain) during cancer treatment. However, although IL-6 did not change during cancer treatment, the symptom cluster was associated with both TNF-α and IL-6, suggesting that only part of the inflammation leading to symptom experience was due to treatment.

In a sample of early stage breast cancer patients followed for 2 years, Starkweather et al. observed an association between increased CRP and reductions in several aspects of cognitive functioning but not between CRP and cancer treatment modality [41]. However, in a follow-up study using the same patient sample, cognitive functioning was associated with treatment modality [42]. Further, CRP was associated with body mass index and education level, both of which also predicted cognitive functioning, thus suggesting that the contribution of CRP to symptom severity was not dependent on treatment, but rather reflective of more stable patient characteristics. This same group also reported that a cluster of symptoms including fatigue was not associated with CRP [41].

Reports are also emerging on associations between genetic polymorphisms of inflammation-regulating genes and symptoms, which could contribute to individual differences in the inflammatory response to cancer treatment. Doong et al. [43••] found that single nucleotide polymorphisms (SNPs) in inflammation-regulating genes *IL6*, *TNFα*, and *IL13* predicted a combination of severe fatigue and pain in patients prior to breast cancer surgery. In this study, more patients with high symptom severity than with low symptom severity had undergone neoadjuvant chemotherapy. Thus, symptom experience was possibly the result of chemotherapy-induced inflammation. In the same sample, SNPs in the *IL1R1* and *IL13* genes were associated with breast pain prior to surgery [16], and SNPs in the *IL1r2* and *IL10* genes were associated with severe and ongoing breast pain after surgery [44]. Kober et al. [45] reported increased prevalence of SNPs in the *IL1b* and *IL10* genes in breast cancer patients who consistently reported high levels of fatigue during a 6-month period. Of note, patients in the high fatigue group more often had undergone neoadjuvant chemotherapy or received chemotherapy during the study. Thus, here again, fatigue was probably caused by chemotherapy-induced inflammation, which might have been exaggerated by a genetic vulnerability for inflammatory responses. In contrast, Chae et al. [46] reported that SNPs in *TNFα* and *IL6* genes were not associated with chemotherapy-related cognitive impairments. We propose that associations between certain SNPs in inflammation-related genes and symptom experience are inconclusive at best. Most likely, higher symptom burden is related to the severity of the cancer treatment. It would be interesting to assess whether a genetic profile fostering a proinflammatory phenotype would identify patients with increased vulnerability to neurotoxic symptom development.

### Neuroinflammation and Symptoms in Survivorship

Although inflammation has been associated with symptoms during survivorship, Smith et al. [39] and others suggest that the peripheral mild increase in inflammatory mediators at this time is not the direct result of previous cancer treatment. However, the findings described above on symptoms and SNPs in inflammatory genes during and shortly after treatment might also explain the associations between inflammation and symptoms during survivorship: these SNPs would imply an enhanced vulnerability for inflammatory responses to new challenges experienced after cancer therapy. For example, Bower et al. [47•] showed that a higher genetic risk index (i.e., presence of alleles associated with higher IL-1β, IL-6, and TNF-α expression) was associated with fatigue and memory complaints in breast cancer patients 3 months after primary treatment. This approach of studying a combination of SNPs already linked to increased expression of proinflammatory mediators provides more insights than studying individual SNPs for which the functionality is often unknown. More studies using this approach will eventually provide a better insight into the possibility of a proinflammatory phenotype as a vulnerability for developing neurotoxic symptoms.

### Changes in Brain Structure and Activity as Mediator of the Association between Neuroinflammation and Symptoms

Evidence is accumulating that cancer therapy leads to changes in brain structure and metabolism that are associated with inflammation and symptoms. Kesler et al. [48] reported an association between lower left hippocampal volume as measured by MRI and higher levels of circulating TNF-α and lower levels of IL-6 in breast cancer survivors, but not in controls. Verbal memory performance in survivors was predicted by an interaction between hippocampal volume and TNF-α. Most of the breast cancer survivors in this study (>80%) had received a chemotherapeutic cocktail including doxorubicin, which was shown in an animal model to increase TNF-α (and IL-1β) in the hippocampus [49]. Thus, the association between *circulating* TNF-α and hippocampal volume found in the clinical study possibly reflects associations between *central* TNF-α and hippocampal volume, leading to cognitive symptoms. A small pilot study that included eight breast cancer patients who underwent chemotherapy during the study [50] found associations of higher sTNFR2 and IL-6 levels with decreased gray matter volume in specific regions. However, fatigue and cognitive functioning were not related to the inflammatory markers. Two preliminary reports from an ongoing longitudinal study showed associations between plasma levels of inflammatory mediators and reduced brain metabolism (as measured by FDG PET-scan) after chemotherapy for breast cancer: Ganz et al. [51] showed an association between plasma sTNFR2 and increased memory complaints, along with *diminished* resting-state metabolism in the inferior frontal regions in a subsample of 12 breast cancer patients who had undergone chemotherapy. Declines in sTNFR2 over the 12-month study period were associated with fewer memory complaints. Interestingly, associations between plasma sTNFR2 and memory complaints diminished when controlled for fatigue, implying that the report of memory complaints was partly due to inflammation-induced fatigue. Conversely, Pomykala [52], reporting on the same longitudinal study but with a larger sample (23 chemotherapy-treated patients), observed associations between higher plasma levels of inflammatory mediators (IL-6, sTNFRII, IL-1ra, and CRP) and *increased* resting-state metabolism in prefrontal and anterior temporal cortex shortly after chemotherapy completion as well as 1 year later. Metabolism in these brain regions was associated with patient-reported memory complaints. These apparently contradictory findings might be explained by a compensatory system in which reduced activity in some brain regions is compensated by increased activity in other regions. Zick et al. [53] found that both IL-6 and specific brain metabolites (higher glutamate + glutamine to N-acetyl-aspartate ratio in the posterior insula) predicted fatigue in breast cancer survivors. However, these brain metabolites did not correlate with IL-6, suggesting that not all changes in brain metabolism are inflammation-related, although we note that only one inflammation marker was tested.

### Summary

Cancer therapy can elicit a peripheral inflammatory response that may reflect central inflammation, and correlations between neurotoxic symptoms and peripheral inflammation markers are consistently found, even long after completion of cancer treatment. Individual genetic differences in the capacity to mount an inflammatory response to environmental challenges might explain who will develop inflammation-related symptoms during and after cancer therapy. However, not all inflammatory mediators associated with symptom development result from cancer therapy, and there is little evidence to suggest that therapy-related inflammatory processes last long after treatment cessation. Thus, other causal mechanisms of inflammation both during and after treatment need to be considered.

Findings on brain structure and metabolism, although sparse and in need of replication, suggest that chemotherapy-induced increases in inflammation, specifically TNF-α or sTNFR2 (and to a lesser extent, IL-6), are associated with changes in brain volume accompanied by decreased brain metabolism in some regions and possibly with compensatory increases in metabolism in other regions. These central changes in volume and metabolism are associated with both cognitive impairment and fatigue.

## Neuroendocrine Function and Symptom Experience

Psychosocial stress, cancer, and cancer treatment all affect neuroendocrine function. Dysregulations in neuroendocrine function are associated with symptom burden via both inflammatory and non-inflammatory pathways.

### Psychosocial Stress

Distress peaks at the time of a breast cancer diagnosis, with 60–77% of patients reporting moderate to severe distress at this point [54, 55] and declines slowly after completion of treatment [56]. Still, up to 40% of patients experience moderate to severe distress 6 months after diagnosis, and 30% report distress 15 months postdiagnosis [57].

Associations between distress and symptom severity are observed before, during, and after completion of cancer therapy [58]. Xiao et al. [59] reported that stress led to fatigue in breast cancer survivors, mostly via the development of depressive symptoms. These effects were independent of the effects of several inflammatory mediators related to fatigue. Several factors have been identified as contributors to a heightened vulnerability for stress and subsequent neurotoxic symptoms in breast cancer patients. For example, neuroticism is a personality characteristic related to high stress vulnerability. Deimling et al. [60] found that neuroticism was the strongest predictor of cancer-related worry in older adult survivors, and Lo-Fo-Wong et al. [57] reported that high stress at both 6 and 15 months postdiagnosis was associated with more frequent cancer worry and higher neuroticism. Neuroticism has also been related to fatigue in breast cancer survivors [61]. Stress during diagnosis of breast cancer was related to younger age and having experienced emotional problems in the past in a study by Jorgensen et al. [54]. Also prior to cancer treatment, Han et al. found an association between childhood trauma and increased expression of gene transcripts related to inflammatory signaling [62].

### Cortisol and Symptoms

Cortisol is released by the hypothalamic-pituitary-adrenal (HPA) axis in response to psychosocial stress. Elevations in cortisol are typically followed by a rapid decline in cortisol production due to feedback mechanisms inhibiting the HPA axis. Stress-related disorders are characterized by sharper acute increases in cortisol in response to a stressor or slower declines after the stressor, indicating a sensitized HPA response or a desensitized feedback loop, respectively [63, 64]. In addition, cortisol concentrations follow a diurnal rhythm, with a sharp increase during awakening followed by a gradual decline during the day. Stress has also been shown to lead to a blunting of the diurnal slope (i.e., reduced decrease in cortisol during the day) [65], which has been associated with, among others, depressive symptoms, fatigue, and pain [66, 67].

Preliminary evidence indicates that tumors can affect endocrine function. Preclinical studies have shown that corticosterone (the primary glucocorticoid in most animals) levels are generally higher in tumor-bearing rodents than in tumor-free controls, and stress-hormone responsivity to stress is reduced in these animals [68•]. Similar observations have been made in patients, although causes of the dysregulations are more difficult to determine and could include a combination of the tumor and psychosocial stress associated with the diagnosis [68•].

Studies of the association between general neuroendocrine functioning and symptoms during cancer treatment suggest that cancer treatment itself does not affect neuroendocrine functioning. For example, Tell et al. [69•] showed that radiation therapy was not a significant predictor of changes in diurnal cortisol rhythm in recently diagnosed breast cancer patients. However, an association between higher fatigue with higher wakening saliva cortisol levels and a flatter slope in diurnal cortisol decline was observed, suggesting that dysregulations in the neuroendocrine stress response occur independently of cancer treatment. Further, Schmidt et al. [70] reported an association between physical fatigue and increased evening cortisol levels as well as higher overall cortisol secretion in a patients at different stages of treatment; the association between cortisol parameters and fatigue was independent of treatment status.

Although primary cancer treatments do not seem to affect neuroendocrine functioning, supportive medication and adjuvant endocrine therapy do. Dexamethasone, a synthetic glucocorticoid often used during chemotherapy, is known to suppress adrenal function [71]. Furthermore, Baumgart et al. [72] reported higher cortisol levels in survivors treated with aromatase inhibitors or tamoxifen. Andreano et al. [73] observed decreased cortisol response to a physical stressor (cold pressor test) in combination with mild impairments in memory performance in nine survivors receiving treatment with the GRnH agonist Lupron. In summary, breast cancer patients show alterations in neuroendocrine functioning that are associated with symptom experience. Whereas primary cancer treatments do not seem responsible for these alterations, supportive medication and endocrine therapy have been shown to alter neuroendocrine function. Observations on alterations occurring very early on, such as reported by Tell et al. [69•], suggest that nontreatment factors such as the tumor itself and stress due to the diagnosis also contribute.

Several studies by Bower et al. indicated that the neuroendocrine system in breast cancer survivors has reduced capacity to regulate inflammatory responses and that fatigue during survivorship is associated with a blunted cortisol response and more-pronounced inflammatory response to psychological stress [74, 75]. Possibly, the lack of cortisol response leads to insufficient control of the inflammatory response. This group also reported a decreased expression of transcripts containing response elements for glucocorticoids on leukocytes, combined with increased expression of genes coding for a proinflammatory reaction [76]. These changes in gene expression would result in a decreased leukocyte sensitivity to the (inhibiting) effects of cortisol, combined with a more pronounced readiness to initiate a proinflammatory response. In line with this, fatigued breast cancer survivors exhibit a flatter diurnal cortisol slope [77], suggesting that the feedback loop for cortisol is also desensitized. A flatter cortisol slope would result in higher daily levels of cortisol, which might ultimately contribute to immune cells being less sensitive to the effects of cortisol. These findings point towards dysregulated neuroendocrine response and decreased capacity of glucocorticoids to regulate the inflammatory response in survivors, both reflecting the effects of chronic exposure to stress hormones induced by stress and cancer treatment.

### Catecholamines, Heart Rate Variability, and Symptoms

Stress also activates the sympathetic nervous system (SNS), leading to enhanced levels of catecholamines such as epinephrine. Catecholamines influence immune activity mostly via activating β2-adrenergic receptors on leukocytes, leading to increases in intracellular cAMP and activation of anti-inflammatory cytokines [78]. However, especially norepinephrine has a high affinity for α-adrenergic receptors which are present on monocytes and macrophages that can convey more proinflammatory signaling [79, 80].

Repeated exposure to catecholamines as a result of chronic stress has been linked to poorer breast cancer outcomes [81, 82•]. However, few studies have directly investigated associations between catecholamines and symptoms in breast cancer patients and survivors. Thornton et al. [83] reported moderate positive associations between epinephrine and symptoms of pain and fatigue in patients with advanced breast cancer.

The *COMT* gene regulates expression of catechol-O-methyltransferase (COMT) enzymes, which metabolize dopamine, norepinephrine, and epinephrine; several SNPs in the *COMT* gene have been associated with symptoms in breast cancer patients and survivors. Functional outcomes of one of these SNPs (i.e., rs4680 or Val158Met) are well characterized: compared to carriers of the Val allele, homozygous Met (Met/Met) carriers have three to four times lower enzyme activity resulting in slower clearance of catecholamines. The homozygous Met genotype has been associated with increased stress vulnerability [84] but better cognitive performance [85] in non-stressful situations [86]. Breast cancer survivors carrying the Met/Met genotype reported higher fatigue and exhibited higher pain sensitivity and higher cortisol levels compared with survivors carrying the more stress-resilient variants [87, 88]. Showing the protective effect of the Met/Met genotype on cognition, Small et al. [89] reported that Met/Met carriers performed better on several measures of cognitive performance and showed an interaction between the genotype and type of cancer therapy: carriers of the Val allele who had received chemotherapy performed more poorly on tests of attention, compared with Val allele carriers who had received radiation. A polymorphism in a different region of the *COMT* gene (rs165599) was associated with less decline in memory performance during chemotherapy [90].

One important outcome of increased activity of the sympathetic nervous system, and thus increased secretion of catecholamines, is decreased heart rate variability (HRV). HRV reflects a balance between sympathetic and parasympathetic activity. The sympathetic nervous system affects the heart rate via release of epinephrine and norepinephrine [91]. Higher catecholamine levels lead to lower HRV, which is associated with poorer health outcomes [91], depression, and fatigue. Lower HRV is also a known outcome of chronic psychosocial stress. Treatment with anthracyclines like doxorubicin, a class of chemotherapeutics commonly used in the treatment of breast cancer, are thought to lower HRV [82•]. Several studies reported an association between lower HRV and fatigue in breast cancer survivors [92]. Fagundes et al. showed a lower resting-state HRV and higher norepinephrine levels in association with higher fatigue in breast cancer survivors. Interestingly, changes in HRV in response to an experimental acute stressor were not associated with fatigue, suggesting that basal secretion of catecholamines, but not catecholaminergic responsiveness, is related to fatigue. Underlining the notion that inflammation is not the only pathway via which stress leads to symptom experience, Crosswell et al. [93] observed an association between lower HRV and higher fatigue in breast cancer survivors but did not find a mediating effect of inflammation in this association.

### Summary

Stress is highly prevalent in breast cancer patients and, especially when prolonged, can lead to dysregulations in neuroendocrine functioning that generally represent as higher overall cortisol levels combined with reduced responsivity to cortisol. Although primary cancer therapies such as chemotherapy and radiation do not seem to alter endocrine function, synthetic glucocorticoids administered during cancer therapy and adjuvant hormonal therapies do have the potential to alter endocrine function. The few studies on catecholaminergic outcomes suggest that increased catecholamine secretion is associated with increased symptom experience.

## Changes in Cellular Bioenergetics Resulting from Cancer Therapy, Inflammation, and Stress

Evidence is emerging that mitochondrial dysfunction is at the origin of several cancer-related symptoms. Mitochondria are responsible for cellular energy production by converting adenosine diphosphate (ADP) into adenosine triphosphate (ATP) through aerobic respiration. This conversion occurs in a series of redox reactions in the electron-transport chain-enzyme compounds, labeled complex I–IV. Changes in any of these complexes create changes in energy output.

Damage to mitochondria, including their own DNA (mtDNA), leads to reduced cellular energy which has been associated with many neurologic symptoms [94]. Mitochondria are especially vulnerable for oxidative stress, a disturbed balance between reactive oxygen species (ROS) and antioxidants. Cancer treatment, inflammation, and stress can affect mitochondrial function by increasing ROS thereby damaging mitochondria [17, 95]. Mitochondrial dysfunction can also lead to overproduction of ROS, inducing a downward spiral in cellular functioning [96].

### Mitochondrial Dysfunction

Several chemoagents have been shown to affect mitochondrial function. Doxorubicin treatment has been associated with impairments in hippocampal mitochondrial enzyme complexes I and II and with redox activity in rats [49]. Cisplatin treatment was shown to decrease neuronal mitochondrial capacity on the level of spare respiratory capacity [97, 98], which translates to reduced capacity to increase energy production when demand increases. Taxanes, often used in breast cancer treatment, were shown to cause structural mitochondrial damage [99]. Other cancer treatment modalities likewise seem to affect mitochondrial function. For example, radiation therapy was shown to increase oxidative stress markers in breast cancer patients with severe acute skin reactions to radiation [100]. Further, maintenance therapeutic agents such as tamoxifen were also shown to negatively affect mitochondrial functioning [101]. Interestingly, in a rodent model of head and neck cancer, chemoradiation seemed to normalize the tumor-related changes in mtDNA expression in the *liver* while causing severe changes in mtDNA expression in the *brain*, suggesting that cancer treatment specifically affects cellular energy production in the central nervous system.

Glucocorticoids, released during stress or administered as part of cancer therapy, can affect mtDNA transcription by binding to mitochondrial GRs [95, 102]. Glucocorticoid effects on mitochondrial energy production seem dose-dependent in a u-shaped manner: exposure to several low doses of glucocorticoids is associated with enhancement of neuronal mitochondrial function, whereas exposure to high doses is associated with a reduction in function [103]. Nonetheless, low-dose glucocorticoid exposure can become detrimental when repeated often enough [104, 105]. Catecholamines, released during stress by the SNS, can also affect mitochondrial function, either by increasing the metabolic state [106, 107], or by inducing ROS production leading to cytogenetic damage [108].

Cancer therapy can lead to long-lasting changes in the central nervous system. For example, Kesler et al. [109] showed alterations in cerebral white-matter organization as assessed with diffusion tensor imaging in chemotherapy-treated breast cancer survivors, along with altered functional connectivity in several brain regions [110, 111]. Furthermore, inflammation has been associated with reductions in brain volume and brain metabolism, as discussed above. We propose that these changes are (in part) the result of reduced cellular activity due to mitochondrial damage.

### Impaired Mitochondrial Function and Neurotoxic Symptoms

Evidence is emerging that cancer treatment-induced neurotoxicities are related to mitochondrial damage and reduced function. Kober et al. [112], comparing breast cancer patients undergoing chemoradiation who did or did not have evening fatigue, showed increased expression in genes related to mitochondrial dysfunction in patients with evening fatigue. Furthermore, radiation-related fatigue was associated with expression of several mitochondrial genes in prostate cancer patients [113].

Animal models provide insight into effects of mitochondrial dysfunction on neurotoxic symptoms. Xiao et al. [114] observed an increase in neuropathic pain after administration of mitochondrial toxins in rats with taxane-induced neuropathy, but not in rats that had not received taxanes, suggesting that taxanes render the mitochondria more vulnerable to further insult. Our group showed that taxane treatment-induced structural damage in mitochondria in the dorsal root ganglia and peripheral nerves of mice [99]. In addition, Zheng et al. [115] showed that both taxanes and platinum-based chemoagents reduce mitochondrial functioning. These changes might be the cause of the nerve-ending retraction from the epidermis observed in animal and clinical studies of chemotherapy-induced neuropathic pain [99, 116, 117]. Indeed, treatment with pifithrin-μ, a small molecule that protects mitochondria by inhibiting mitochondrial p53 accumulation and thereby preventing activation of mitochondrial damage pathways [118], not only prevented the taxane-induced mitochondrial damage and neuropathic pain in mice, but also nerve fiber loss in the epidermis [99].

Our lab also observed that cisplatin-induced cognitive impairments are accompanied by structural changes in mitochondria and decreased mitochondrial function in brain synaptosomes in mice [98]. Unchallenged (basal) mitochondrial function was comparable to that seen in control mice, suggesting that only the mitochondrial capacity to increase energy production in more demanding situations was impaired. In addition, cisplatin decreased the number of neuronal progenitors in specific brain areas and reduced white-matter coherency, as has also been observed in humans via neuroimaging. The mitochondrial protectant pifithrin-μ prevented not only mitochondrial damage and cognitive impairments, but also cisplatin-induced changes in neuronal precursors and white-matter integrity, suggesting that these changes are downstream consequences of cisplatin-induced mitochondrial dysfunction. Lomelli reported similar findings for cisplatin-treated rats [97].

We propose that cancer therapy-induced mitochondrial damage leads to changes in brain structure and functional integrity, resulting in enhanced energy demands during cognitive challenges. However, this increased demand cannot be met by the mitochondria, leading to impaired performance on challenging cognitive tasks.

## Conclusion

The association between neuroinflammation and breast cancer-related neurotoxicities is well-established. Associations between inflammatory markers and neurotoxicities seem to exist even when inflammation is low. Possibly, inflammation is only an important mediator in individuals with a genetic vulnerability for exaggerated inflammatory responses to internal (tumor, stress) and external (cancer therapy) stressors.

Stress is highly prevalent in breast cancer patients. Although stress peaks during diagnosis and declines during treatment, individual characteristics, such as a tendency to worry or childhood trauma, might lead to prolonged stress in a subset of patients. Prolonged stress diminishes the capacity of stress hormones to regulate the inflammatory response, resulting in exaggerated cytokine and ROS production. Stress could also lead to symptom experience independent of inflammation.

We suggest that mitochondrial dysfunction is a final common outcome of cancer, cancer therapy, inflammation/ROS, and stress that leads to neurotoxic symptoms. Recent evidence for mitochondria-protecting drugs preventing cancer therapy-related toxicities points to promising avenues for treatment of neurotoxicities in breast cancer patients. Establishment of these drugs in clinical settings, as well as early implementation of stress-reduction interventions (during or shortly after diagnosis) should be considered to prevent the long-term neurotoxic symptoms that plague so many breast cancer survivors.

## References

[1] Servaes P, Verhagen C, Bleijenberg G (2002). Fatigue in cancer patients during and after treatment: prevalence, correlates and interventions. Eur J Cancer.

[2] Frank JS, Vance DE, Triebel KL, Meneses KM (2015). Cognitive deficits in breast cancer survivors after chemotherapy and hormonal therapy. J Neurosci Nurs.

[3] Bao T, Basal C, Seluzicki C, Li SQ, Seidman AD, Mao JJ (2016). Long-term chemotherapy-induced peripheral neuropathy among breast cancer survivors: prevalence, risk factors, and fall risk. Breast Cancer Res Treat.

[4] Bhatnagar B, Gilmore S, Goloubeva O, Pelser C, Medeiros M, Chumsri S (2014). Chemotherapy dose reduction due to chemotherapy induced peripheral neuropathy in breast cancer patients receiving chemotherapy in the neoadjuvant or adjuvant settings: a single-center experience. Spring.

[5] Rashid N, Koh HA, Baca HC, Li Z, Malecha S, Abidoye O (2015). Clinical impact of chemotherapy-related adverse events in patients with metastatic breast cancer in an integrated health care system. J Manag Care Spec Pharm.

[6] Tevaarwerk AJ, Lee JW, Sesto ME, Buhr KA, Cleeland CS, Manola J (2013). Employment outcomes among survivors of common cancers: the symptom outcomes and practice patterns (SOAPP) study. J Cancer Surviv.

[7] Society AC (2015). Breast cancer facts & figures 2015–2016.

[8] Jim HS, Phillips KM, Chait S, Faul LA, Popa MA, Lee YH (2012). Meta-analysis of cognitive functioning in breast cancer survivors previously treated with standard-dose chemotherapy. J Clin Oncol.

[9] Taillibert S, Le Rhun E, Chamberlain MC. Chemotherapy-related neurotoxicity. Curr Neurol Neurosci Rep. 2016;16(9). doi:10.1007/s11910-016-0686-x. 10.1007/s11910-016-0686-x 27443648

[10] Bower JE, Ganz PA (2015). Symptoms: fatigue and cognitive dysfunction. Adv Exp Med Biol.

[11] Bower JE, Ganz PA, May LT, Hu W, Belin TR, Sepah S (2009). Inflammatory biomarkers and fatigue during radiation therapy for breast and prostate cancer. Clin Cancer Res.

[12] De Sanctis V, Agolli L, Visco V, Monaco F, Muni R, Spagnoli A, et al. Cytokines, fatigue, and cutaneous erythema in early stage breast cancer patients receiving adjuvant radiation therapy. Biomed Res Int. 2014;2014 doi:10.1155/2014/523568. 10.1155/2014/523568 PMC3988916 24800238

[13] Starkweather AR, Lyon DE, Schubert CM (2013). Pain and inflammation in women with early-stage breast cancer prior to induction of chemotherapy. Biol Res Nurs.

[14] Albuquerque K, Tell D, Lobo P, Millbrandt L, Mathews HL, Janusek LW (2012). Impact of partial versus whole breast radiation therapy on fatigue, perceived stress, quality of life and natural killer cell activity in women with breast cancer. BMC Cancer.

[15] Joly F, Giffard B, Rigal O, De Ruiter MB, Small BJ, Dubois M (2015). Impact of cancer and its treatments on cognitive function: advances in research from the Paris International Cognition and Cancer Task Force symposium and update since 2012. J Pain Symptom Manag.

[16] McCann B, Miaskowski C, Koetters T, Baggott C, West C, Levine JD (2012). Associations between pro- and anti-inflammatory cytokine genes and breast pain in women prior to breast cancer surgery. J Pain.

[17] Vichaya EG, Chiu GS, Krukowski K, Lacourt TE, Kavelaars A, Dantzer R (2015). Mechanisms of chemotherapy-induced behavioral toxicities. Front Neurosci.

[18] Cleeland CS, Bennett GJ, Dantzer R, Dougherty PM, Dunn AJ, Meyers CA (2003). Are the symptoms of cancer and cancer treatment due to a shared biologic mechanism? A cytokine-immunologic model of cancer symptoms. Cancer.

[19] Miller AH, Ancoli-Israel S, Bower JE, Capuron L, Irwin MR (2008). Neuroendocrine-immune mechanisms of behavioral comorbidities in patients with cancer. J Clin Oncol.

[20] Carlson LE, Waller A, Groff SL, Giese-Davis J, Bultz BD (2013). What goes up does not always come down: patterns of distress, physical and psychosocial morbidity in people with cancer over a one year period. Psychooncology.

[21] van Zuiden M, Kavelaars A, Vermetten E, Olff M, Geuze E, Heijnen C (2015). Pre-deployment differences in glucocorticoid sensitivity of leukocytes in soldiers developing symptoms of PTSD, depression or fatigue persist after return from military deployment. Psychoneuroendocrinology.

[22] Mausbach BT, Aschbacher K, Mills PJ, Roepke SK, von Kanel R, Patterson TL (2008). A 5-year longitudinal study of the relationships between stress, coping, and immune cell beta(2)-adrenergic receptor sensitivity. Psychiatry Res.

[23] Reul JMHM, Collins A, Saliba RS, Mifsud KR, Carter SD, Gutierrez-Mecinas M (2015). Glucocorticoids, epigenetic control and stress resilience. Neurobiology of Stress.

[24] Saligan LN, Kim HS (2012). A systematic review of the association between immunogenomic markers and cancer-related fatigue. Brain Behav Immun.

[25] Wang XM, Walitt B, Saligan L, Tiwari AFY, Cheung CW, Zhang ZJ (2015). Chemobrain: a critical review and causal hypothesis of link between cytokines and epigenetic reprogramming associated with chemotherapy. Cytokine.

[26] •• Patel SK, Wong AL, Wong FL, Breen EC, Hurria A, Smith M et al. Inflammatory biomarkers, comorbidity, and neurocognition in women with newly diagnosed breast cancer. J Natl Cancer Inst. 2015;107(8). doi:10.1093/jnci/djv131. **Study including large sample of breast cancer patients investigating the presence of cancer-related symptoms prior to onset of any cancer treatment and the association between symptoms and inflammation. Unique in its objective to study symptoms in treatment-naïve patients.** 10.1093/jnci/djv131 PMC4609551 26101331

[27] Wang L, Chen Q, Qi H, Wang C, Wang C, Zhang J (2016). Doxorubicin-induced systemic inflammation is driven by upregulation of toll-like receptor TLR4 and endotoxin leakage. Cancer Res.

[28] Fitzpatrick FA, Wheeler R (2003). The immunopharmacology of paclitaxel (Taxol®), docetaxel (Taxotere®), and related agents. Int Immunopharmacol.

[29] Vyas D, Laput G, Vyas AK (2014). Chemotherapy-enhanced inflammation may lead to the failure of therapy and metastasis. Onco Targets Ther.

[30] Boomsma MF, Garssen B, Slot E, Berbee M, Berkhof J, Meezenbroek Ede J (2010). Breast cancer surgery-induced immunomodulation. J Surg Oncol.

[31] Schaue D, McBride WH (2010). Links between innate immunity and normal tissue radiobiology. Radiat Res.

[32] Burnette B, Weichselbaum RR (2013). Radiation as an immune modulator. Semin Radiat Oncol.

[33] Pertl MM, Hevey D, Boyle NT, Hughes MM, Collier S, O'Dwyer AM (2013). C-reactive protein predicts fatigue independently of depression in breast cancer patients prior to chemotherapy. Brain Behav Immun.

[34] Schmidt ME, Meynköhn A, Habermann N, Wiskemann J, Oelmann J, Hof H (2016). Resistance exercise and inflammation in breast cancer patients undergoing adjuvant radiation therapy: mediation analysis from a randomized, controlled intervention trial. International Journal of Radiation Oncology Biology Physics.

[35] Liu L, Mills PJ, Rissling M, Fiorentino L, Natarajan L, Dimsdale JE (2012). Fatigue and sleep quality are associated with changes in inflammatory markers in breast cancer patients undergoing chemotherapy. Brain Behav Immun.

[36] Raudonis BM, Kelley IH, Rowe N, Ellis J (2016). A pilot study of proinflammatory cytokines and fatigue in women with breast cancer during chemotherapy. Cancer Nurs.

[37] Cheung YT, Ng T, Shwe MK, Ho H, Foo KM, Cham MT (2015). Association of proinflammatory cytokines and chemotherapy-associated cognitive impairment in breast cancer patients: a multi-centered, prospective, cohort study. Ann Oncol.

[38] Shibayama O, Yoshiuchi K, Inagaki M, Matsuoka Y, Yoshikawa E, Sugawara Y (2014). Association between adjuvant regional radiotherapy and cognitive function in breast cancer patients treated with conservation therapy. Cancer medicine.

[39] Smith AK, Conneely KN, Pace TWW, Mister D, Felger JC, Kilaru V (2014). Epigenetic changes associated with inflammation in breast cancer patients treated with chemotherapy. Brain Behav Immun.

[40] Reed RG, Weihs KL, Sbarra DA, Breen EC, Irwin MR, Butler EA (2016). Emotional acceptance, inflammation, and sickness symptoms across the first two years following breast cancer diagnosis. Brain Behav Immun.

[41] Starkweather A, Kelly DL, Thacker L, Wright ML, Jackson-Cook CK, Lyon DE (2017). Relationships among psychoneurological symptoms and levels of C-reactive protein over 2 years in women with early-stage breast cancer. Support Care Cancer.

[42] Lyon DE, Cohen R, Chen H, Kelly DL, Starkweather A, Ahn HC (2016). The relationship of cognitive performance to concurrent symptoms, cancer- and cancer-treatment-related variables in women with early-stage breast cancer: a 2-year longitudinal study. J Cancer Res Clin Oncol.

[43] Doong SH, Dhruva A, Dunn LB, West C, Paul SM, Cooper BA (2015). Associations between cytokine genes and a symptom cluster of pain, fatigue, sleep disturbance, and depression in patients prior to breast cancer surgery. Biol Res Nurs.

[44] Stephens K, Cooper BA, West C, Paul SM, Baggott CR, Merriman JD (2014). Associations between cytokine gene variations and severe persistent breast pain in women following breast cancer surgery. J Pain.

[45] Kober KM, Smoot B, Paul SM, Cooper BA, Levine JD, Miaskowski C (2016). Polymorphisms in cytokine genes are associated with higher levels of fatigue and lower levels of energy in women after breast cancer surgery. J Pain Symptom Manag.

[46] Chae JW, Ng T, Yeo HL, Shwe M, Gan YX, Ho HK et al. Impact of TNF-α (rs1800629) and IL-6 (rs1800795) polymorphisms on cognitive impairment in Asian breast cancer patients. PLoS One. 2016;11(10). doi:10.1371/journal.pone.0164204. 10.1371/journal.pone.0164204 PMC5049844 27701469

[47] Bower JE, Ganz PA, Irwin MR, Castellon S, Arevalo J, Cole SW (2013). Cytokine genetic variations and fatigue among patients with breast cancer. Journal of clinical oncology : official journal of the American Society of Clinical Oncology.

[48] Kesler S, Janelsins M, Koovakkattu D, Palesh O, Mustian K, Morrow G (2013). Reduced hippocampal volume and verbal memory performance associated with interleukin-6 and tumor necrosis factor-alpha levels in chemotherapy-treated breast cancer survivors. Brain Behav Immun.

[49] Kwatra M, Jangra A, Mishra M, Sharma Y, Ahmed S, Ghosh P (2016). Naringin and sertraline ameliorate doxorubicin-induced behavioral deficits through modulation of serotonin level and mitochondrial complexes protection pathway in rat hippocampus. Neurochem Res.

[50] Jenkins V, Thwaites R, Cercignani M, Sacre S, Harrison N, Whiteley-Jones H et al. A feasibility study exploring the role of pre-operative assessment when examining the mechanism of ‘chemo-brain’ in breast cancer patients. SpringerPlus. 2016;5(1). doi:10.1186/s40064-016-2030-y. 10.1186/s40064-016-2030-y PMC4816933 27047716

[51] Ganz PA, Bower JE, Kwan L, Castellon SA, Silverman DHS, Geist C et al. Does tumor necrosis factor-alpha (TNF-α) play a role in post-chemotherapy cerebral dysfunction? Brain Behav Immun. 2013;30(SUPPL):S99-S108. doi:10.1016/j.bbi.2012.07.015. 10.1016/j.bbi.2012.07.015 PMC3522786 22884417

[52] Pomykala KL, Ganz PA, Bower JE, Kwan L, Castellon SA, Mallam S (2013). The association between pro-inflammatory cytokines, regional cerebral metabolism, and cognitive complaints following adjuvant chemotherapy for breast cancer. Brain Imaging Behav.

[53] Zick SM, Zwickey H, Wood L, Foerster B, Khabir T, Wright B (2014). Preliminary differences in peripheral immune markers and brain metabolites between fatigued and non-fatigued breast cancer survivors: a pilot study. Brain Imaging and Behavior.

[54] Jørgensen L, Laursen BS, Garne JP, Sherman KA, Søgaard M (2016). Prevalence and predictors of distress in women taking part in surgical continuity of care for breast cancer: a cohort study. Eur J Oncol Nurs.

[55] Mertz BG, Bistrup PE, Johansen C, Dalton SO, Deltour I, Kehlet H (2012). Psychological distress among women with newly diagnosed breast cancer. Eur J Oncol Nurs.

[56] Lester J, Crosthwaite K, Stout R, Jones RN, Holloman C, Shapiro C (2015). Women with breast cancer: self-reported distress in early survivorship. Oncol Nurs Forum.

[57] Lo-Fo-Wong DNN, de Haes HCJM, Aaronson NK, van Abbema DL, den Boer MD, van Hezewijk M (2016). Predictors of enduring clinical distress in women with breast cancer. Breast Cancer Res Treat.

[58] Myers JS, Wick JA, Klemp J (2015). Potential factors associated with perceived cognitive impairment in breast cancer survivors. Support Care Cancer.

[59] Xiao C, Miller AH, Felger J, Mister D, Liu T, Torres MA. Depressive symptoms and inflammation are independent risk factors of fatigue in breast cancer survivors. Psychol Med. 2017:1–11. doi:10.1017/s0033291717000150. 10.1017/S0033291717000150 28193310

[60] Deimling GT, Albitz C, Monnin K, Renzhofer Pappada HT, Nalepa E, Laroco Boehm M, et al. Personality and psychological distress among older adult, long-term cancer survivors. J Psychosoc Oncol. 2016:1–15. doi:10.1080/07347332.2016.1225145. 10.1080/07347332.2016.1225145 27541961

[61] Wang SH, He GP, Jiang PL, Tang LL, Feng XM, Zeng C (2013). Relationship between cancer-related fatigue and personality in patients with breast cancer after chemotherapy. Psychooncology.

[62] Han TJ, Felger JC, Lee A, Mister D, Miller AH, Torres MA (2016). Association of childhood trauma with fatigue, depression, stress, and inflammation in breast cancer patients undergoing radiotherapy. Psychooncology.

[63] Morris MC, Compas BE, Garber J (2012). Relations among posttraumatic stress disorder, comorbid major depression, and HPA function: a systematic review and meta-analysis. Clin Psychol Rev.

[64] Belvederi Murri M, Pariante C, Mondelli V, Masotti M, Atti AR, Mellacqua Z (2014). HPA axis and aging in depression: systematic review and meta-analysis. Psychoneuroendocrinology.

[65] Sjors A, Ljung T, Jonsdottir IH (2014). Diurnal salivary cortisol in relation to perceived stress at home and at work in healthy men and women. Biol Psychol.

[66] Raison CL, Borisov AS, Woolwine BJ, Massung B, Vogt G, Miller AH (2010). Interferon-alpha effects on diurnal hypothalamic-pituitary-adrenal axis activity: relationship with proinflammatory cytokines and behavior. Mol Psychiatry.

[67] Generaal E, Vogelzangs N, Macfarlane GJ, Geenen R, Smit JH, Penninx BW (2014). Reduced hypothalamic-pituitary-adrenal axis activity in chronic multi-site musculoskeletal pain: partly masked by depressive and anxiety disorders. BMC Musculoskelet Disord.

[68] Pyter LM (2016). The influence of cancer on endocrine, immune, and behavioral stress responses. Physiol Behav.

[69] Tell D, Mathews HL, Janusek LW (2014). Day-to-day dynamics of associations between sleep, napping, fatigue, and the cortisol diurnal rhythm in women diagnosed as having breast cancer. Psychosom Med.

[70] Schmidt ME, Semik J, Habermann N, Wiskemann J, Ulrich CM, Steindorf K (2016). Cancer-related fatigue shows a stable association with diurnal cortisol dysregulation in breast cancer patients. Brain Behav Immun.

[71] Han HS, Park JC, Park SY, Lee KT, Bae SB, Kim HJ (2015). A prospective multicenter study evaluating secondary adrenal suppression after antiemetic dexamethasone therapy in cancer patients receiving chemotherapy: a Korean south West oncology group study. Oncologist.

[72] Baumgart J, Nilsson K, Stavreus Evers A, Kunovac Kallak T, Kushnir MM, Bergquist J (2014). Androgen levels during adjuvant endocrine therapy in postmenopausal breast cancer patients. Climacteric: the journal of the International Menopause Society.

[73] Andreano JM, Waisman J, Donley L, Cahill L (2012). Effects of breast cancer treatment on the hormonal and cognitive consequences of acute stress. Psychooncology.

[74] Bower JE, Ganz PA, Aziz N (2005). Altered cortisol response to psychologic stress in breast cancer survivors with persistent fatigue. Psychosom Med.

[75] Bower JE, Ganz PA, Aziz N, Olmstead R, Irwin MR, Cole SW (2007). Inflammatory responses to psychological stress in fatigued breast cancer survivors: relationship to glucocorticoids. Brain Behav Immun.

[76] Bower JE, Ganz PA, Irwin MR, Arevalo JMG, Cole SW (2011). Fatigue and gene expression in human leukocytes: increased NF-κB and decreased glucocorticoid signaling in breast cancer survivors with persistent fatigue. Brain Behav Immun.

[77] Bower JE, Ganz PA, Dickerson SS, Petersen L, Aziz N, Fahey JL (2005). Diurnal cortisol rhythm and fatigue in breast cancer survivors. Psychoneuroendocrinology.

[78] Kolmus K, Tavernier J, Gerlo S (2015). beta2-adrenergic receptors in immunity and inflammation: stressing NF-kappaB. Brain Behav Immun.

[79] Elenkov IJ, Wilder RL, Chrousos GP, Vizi ES (2000). The sympathetic nerve—an integrative interface between two supersystems: the brain and the immune system. Pharmacol Rev.

[80] Rouppe van der Voort C, Kavelaars A, van de Pol M, Heijnen CJ (1999). Neuroendocrine mediators up-regulate alpha1b- and alpha1d-adrenergic receptor subtypes in human monocytes. J Neuroimmunol.

[81] Childers WK, Hollenbeak CS, Cheriyath P (2015). Beta-blockers reduce breast cancer recurrence and breast cancer death: a meta-analysis. Clin Breast Cancer.

[82] Lakoski SG, Jones LW, Krone RJ, Stein PK, Scott JM (2015). Autonomic dysfunction in early breast cancer: incidence, clinical importance, and underlying mechanisms. Am Heart J.

[83] Thornton LM, Andersen BL, Blakely WP (2010). The pain, depression, and fatigue symptom cluster in advanced breast cancer: covariation with the hypothalamic-pituitary-adrenal axis and the sympathetic nervous system. Health Psychol.

[84] Hernaus D, Collip D, Lataster J, Ceccarini J, Kenis G, Booij L et al. COMT Val(158)Met genotype selectively alters prefrontal F-18 fallypride displacement and subjective feelings of stress in response to a psychosocial stress challenge. PLoS One. 2013;8(6). doi:10.1371/journal.pone.0065662. 10.1371/journal.pone.0065662 PMC3683024 23799032

[85] Aguilera M, Barrantes-Vidal N, Arias B, Moya J, Villa H, Ibanez MI (2008). Putative role of the COMT gene polymorphism (Val158Met) on verbal working memory functioning in a healthy population. Am J Med Genet B Neuropsychiatr Genet.

[86] Buckert M, Kudielka BM, Reuter M, Fiebach CJ (2012). The COMT Val158Met polymorphism modulates working memory performance under acute stress. Psychoneuroendocrinology.

[87] Fernández-de-las-Peñas C, Cantarero-Villanueva I, Fernández-Lao C, Ambite-Quesada S, Díaz-Rodríguez L, Rivas-Martínez I (2012). Influence of catechol-o-methyltransferase genotype (Val158Met) on endocrine, sympathetic nervous and mucosal immune systems in breast cancer survivors. Breast.

[88] Fernández-De-Las-Penas C, Fernández-Lao C, Cantarero-Villanueva I, Ambite-Quesada S, Rivas-Martínez I, Del Moral-Avila R (2012). Catechol-O-methyltransferase genotype (Val158met) modulates cancer-related fatigue and pain sensitivity in breast cancer survivors. Breast Cancer Res Treat.

[89] Small BJ, Rawson KS, Walsh E, Jim HS, Hughes TF, Iser L (2011). Catechol-O-methyltransferase genotype modulates cancer treatment-related cognitive deficits in breast cancer survivors. Cancer.

[90] Cheng H, Li W, Gan C, Zhang B, Jia Q, Wang K (2016). The COMT (rs165599) gene polymorphism contributes to chemotherapy-induced cognitive impairment in breast cancer patients. Am J Transl Res.

[91] Task force of the European Society of Cardiology and the north American Society of Pacing and Electrophysiology (1996). Heart rate variability: standards of measurement, physiological interpretation and clinical use. Circulation.

[92] Arab C, Dias DP, Barbosa RT, Carvalho TD, Valenti VE, Crocetta TB (2016). Heart rate variability measure in breast cancer patients and survivors: a systematic review. Psychoneuroendocrinology.

[93] Crosswell AD, Lockwood KG, Ganz PA, Bower JE (2014). Low heart rate variability and cancer-related fatigue in breast cancer survivors. Psychoneuroendocrinology.

[94] Parikh S (2010). The neurologic manifestations of mitochondrial disease. Developmental disabilities research reviews.

[95] Picard M, Juster RP, McEwen BS (2014). Mitochondrial allostatic load puts the 'gluc' back in glucocorticoids. Nat Rev Endocrinol.

[96] Zorov DB, Juhaszova M, Sollott SJ (2014). Mitochondrial reactive oxygen species (ROS) and ROS-induced ROS release. Physiol Rev.

[97] Lomeli N, Di K, Czerniawski J, Guzowski JF, Bota DA (2017). Cisplatin-induced mitochondrial dysfunction is associated with impaired cognitive function in rats. Free Radic Biol Med.

[98] Chiu GS, Maj MA, Rizvi S, Dantzer R, Vichaya EG, Laumet G (2016). Pifithrin-micro prevents cisplatin-induced chemobrain by preserving neuronal mitochondrial function. Cancer Res.

[99] Krukowski K, Nijboer CH, Huo X, Kavelaars A, Heijnen CJ (2015). Prevention of chemotherapy-induced peripheral neuropathy by the small-molecule inhibitor pifithrin-μ. Pain.

[100] Skiold S, Naslund I, Brehwens K, Andersson A, Wersall P, Lidbrink E (2013). Radiation-induced stress response in peripheral blood of breast cancer patients differs between patients with severe acute skin reactions and patients with no side effects to radiotherapy. Mutat Res.

[101] Cardoso CMP, Custódio JBA, Almeida LM, Moreno AJM (2001). Mechanisms of the deleterious effects of tamoxifen on mitochondrial respiration rate and phosphorylation efficiency. Toxicol Appl Pharmacol.

[102] Jeanneteau F, Arango-Lievano M (2016). Linking mitochondria to synapses: new insights for stress-related neuropsychiatric disorders. Neural Plast.

[103] Du J, Wang Y, Hunter R, Wei Y, Blumenthal R, Falke C (2009). Dynamic regulation of mitochondrial function by glucocorticoids. Proc Natl Acad Sci U S A.

[104] Gong Y, Chai Y, Ding JH, Sun XL, Hu G (2011). Chronic mild stress damages mitochondrial ultrastructure and function in mouse brain. Neurosci Lett.

[105] Hunter RG, Seligsohn M, Rubin TG, Griffiths BB, Ozdemir Y, Pfaff DW (2016). Stress and corticosteroids regulate rat hippocampal mitochondrial DNA gene expression via the glucocorticoid receptor. Proc Natl Acad Sci U S A.

[106] Grip J, Jakobsson T, Hebert C, Klaude M, Sandstrom G, Wernerman J (2015). Lactate kinetics and mitochondrial respiration in skeletal muscle of healthy humans under influence of adrenaline. Clin Sci (Lond).

[107] Costa VM, Silva R, Tavares LC, Vitorino R, Amado F, Carvalho F (2009). Adrenaline and reactive oxygen species elicit proteome and energetic metabolism modifications in freshly isolated rat cardiomyocytes. Toxicology.

[108] Djelic N, Radakovic M, Spremo-Potparevic B, Zivkovic L, Bajic V, Stevanovic J (2015). Evaluation of cytogenetic and DNA damage in human lymphocytes treated with adrenaline in vitro. Toxicol in Vitro.

[109] Kesler SR, Watson CL, Blayney DW (2015). Brain network alterations and vulnerability to simulated neurodegeneration in breast cancer. Neurobiol Aging.

[110] Hosseini SMH, Kesler SR (2014). Multivariate pattern analysis of fMRI in breast cancer survivors and healthy women. J Int Neuropsychol Soc.

[111] Kesler SR, Blayney DW (2016). Neurotoxic effects of anthracycline- vs nonanthracycline-based chemotherapy on cognition in breast cancer survivors. JAMA oncology.

[112] Kober KM, Dunn L, Mastick J, Cooper B, Langford D, Melisko M (2016). Gene expression profiling of evening fatigue in women undergoing chemotherapy for breast cancer. Biol Res Nurs..

[113] Hsiao CP, Wang D, Kaushal A, Chen MK, Saligan L (2014). Differential expression of genes related to mitochondrial biogenesis and bioenergetics in fatigued prostate cancer men receiving external beam radiation therapy. J Pain Symptom Manag.

[114] Xiao WH, Bennett GJ (2012). Effects of mitochondrial poisons on the neuropathic pain produced by the chemotherapeutic agents, paclitaxel and oxaliplatin. Pain.

[115] Zheng H, Xiao WH, Bennett GJ (2011). Functional deficits in peripheral nerve mitochondria in rats with paclitaxel- and oxaliplatin-evoked painful peripheral neuropathy. Exp Neurol.

[116] Mao-Ying QL, Kavelaars A, Krukowski K, Huo XJ, Zhou W, Price TJ (2014). The anti-diabetic drug metformin protects against chemotherapy-induced peripheral neuropathy in a mouse model. PLoS One.

[117] Boyette-Davis JA, Walters ET, Dougherty PM (2015). Mechanisms involved in the development of chemotherapy-induced neuropathy. Pain management.

[118] Nijboer CH, Heijnen CJ, van der Kooij MA, Zijlstra J, van Velthoven CT, Culmsee C (2011). Targeting the p53 pathway to protect the neonatal ischemic brain. Ann Neurol.

